# Impact of Processing and Extraction on the Minor Components of Green Spanish-Style Gordal Table Olive Fat, as Assessed by Innovative Approaches

**DOI:** 10.3390/foods9121907

**Published:** 2020-12-20

**Authors:** Antonio López-López, Amparo Cortés-Delgado, Antonio Garrido-Fernández

**Affiliations:** Instituto de la Grasa (CSIC), Food Biotechnology Department, Campus Universitario Pablo de Olavide, Edificio 46, Ctra. Utrera, km 1, 41013 Sevilla, Spain; acortes@cica.es (A.C.-D.); garfer@cica.es (A.G.-F.)

**Keywords:** green Spanish-style Gordal table olives, processing, fat minor components, chemometrics, compositional data analysis, multiple factor analysis

## Abstract

This work aims to study the effect of the green Spanish-style table olive processing and extraction method of fat on its minor components. For this purpose, it uses standard multivariate analysis (developed for Euclidean space), Compositional Data (CoDa) analysis (for data in the simplex) and Multiple Factor analysis (MFA). Overall, processing had a scarce effect on most of the minor components except ethyl and methyl esters and diacylglycerols, which markedly increased during fermentation; however, these compounds in table olive do not have the negative connotations that those in olive oil do since they are normal metabolites from the yeast microflora habitually present during the process. The work also showed that the quantification of minor components in table olive fat was an extraction-dependent method since Soxhlet increased the concentrations of fatty alcohols, triterpene dialcohols, sterols, waxes and polar compounds. Regarding statistical methods, CoDa analysis strategies were successfully applied to produce more appropriate clustering and Principal Component Analysis (PCA) segregation than standard tools. Moreover, MFA allowed for study of the components individually and by groups; the relationships among groups led to the most appropriate clustering and PCA segregation of samples and revealed the effect of the chemical groups’ evolution on the similarity/dissimilarity between samples. Therefore, MFA was the statistical analysis that led to the most information on the effect of processing and extraction methods. Its combination with appropriate CoDa logratios could be an exciting challenge.

## 1. Introduction

Table olive is the major fermented vegetable in western countries. Its production reached 2,500,000 tonnes in the 2017–2018 season [1]. Among them, green Spanish-style table olives are the most popular presentation [2]. Within this style, because of its size (approximately 13 g/fruit), Gordal is suitable to prepare presentations like olives stuffed with cucumber, almond or onion. Their products are about 33,000 tonnes/year in Spain, but other countries are also significant contributors (e.g., Egypt) [3].

Triacylglycerols and their related fatty acids make up most of the table olive lipids [4,5,6,7]. Nevertheless, diverse minor components (sterols, fatty alcohols, triterpenic dialcohols, waxes, fatty acid esters and products of degradation) are still present in these fats. Their concentrations in olive oil may depend on the cultivar, land, climate or adulterations [8]. Among them, sterols, triterpenic dialcohols, waxes and alkyl esters are also used to test the purity and classification of olive oils [9,10]. In table olive flesh, the fat is inside the cells and is hardly adulterated with other oils, but processing might do unexpected and undesirable transformations. Thus, modification of the minor components could be suitable indexes to reflect the aggressiveness of the distinct elaboration procedures and handling operations.

Recent studies have shown that ripe olive processing may affect the olive fat composition [11], including the minor components [12]. The effects were associated with the lipolytic microflora present during the storage/fermentation phase. However, these activities in real green Spanish-style fermentations were limited [13]. The primary nutrient in Gordal presentations is also fat [2], but its content is lower than in other cultivars [4]. A recent study showed that Spanish-style processing produced moderate changes in Gordal fatty acid composition [6,7]. However, the elaboration effect on these minor components is still unknown.

The combination of analytical techniques of minor components and chemometrics has been exploited to differentiate Quercus species and their origins [14] or olive oils from diverse cultivars and growing zones [15]. Table olive fat analysis and chemometrics have been used to identify the effects of ripe olive processing [11,12] or to segregate the oils released during table olive pitting and stuffing [16]. However, these investigations applied standard multivariate statistics, but recently, the use of compositional data analysis (CoDa) integrated a battery of exploratory tools in the study of table olive fatty acids [6,7]. A similar approach could also apply to olive fat minor components. Besides, Multiple Factor Analysis (MFA), which allows for testing of the variables both independently and by group, could be a potential tool scarcely used in chemometrics yet [17].

This work deals with the effect of green Spanish-style processing and extraction methods on the minor components (sterols, fatty alcohols, triterpenic dialcohols, waxes, alkyl esters and polar compounds) of Gordal olive fat. The data were studied by General Linear Model (GLM) as well as by standard multivariate unsupervised (hierarchical clustering and principal component analysis) and were supervised (discriminant analysis), applied to both original values and CoDa isometric logratio (ilr) coordinates. The data set was also analysed using MFA. Finally, we compare the results from the different approaches.

## 2. Materials and Methods

### 2.1. Olives

The fruits, the Gordal cultivar harvested by hand in the middle of September, were supplied by JOLCA SL (Huevar, Sevilla, Spain).

### 2.2. Processing

The olives were processed in duplicate, according to the green Spanish-style, utilizing fibreglass fermenters (21 kg olives and 14 L solution). We performed the debittering process with an 18 g/L NaOH solution, which penetrated 2/3 of the flesh; washed (18 h) the alkali excess with tap water; and finally immersed the fruits in a 110 g NaCl /L brine solution for fermentation. In this brine, the olives followed a spontaneous lactic process in the pilot plant facilities at Instituto de la Grasa (Sevilla). The fermentation was controlled by periodical analysis of the physicochemical characteristics of brines using standard methods [2]. After eight months, 10 kg of fermented olives from each replicate were packaged in glass containers using cover brine analogous to that employed by the industry [2]. The packages were pasteurized (15 min at 80 °C) and stored at room temperature (22 ± 2 °C) for two months before analysis. Therefore, the processing phases, packaging and final product storage were comparable to those applied at an industrial scale.

### 2.3. Samples and Fat Extraction

Samples for fat extraction (approx. 5 kg olives) were withdrawn in duplicate from (i) the fresh Gordal (GT0), (ii) each of the replicates of the fermented fruits (GT2) and (iii) packaged olives (GT3), all of them extracted by Abencor. Besides, another aliquot of fermented (GT2S) and packaged (GT3S) olives were extracted by Soxhlet (S). Each sample consisted of about 1250 olives; therefore, the oils extracted from these fruits (ten in total) were composite samples able to reflect the transformations induced by the different processing phases and extraction methods.

For the extraction by ABENCOR, the fruits were pitted and mixed with a homogenizer Ultraturrax T25 (IKA-Labortecnik, Staufen, Deutschland). Then, the paste was added to warm water reaching about 30 °C in the suspension and the mixture was subjected to malaxation for 40 min at room temperature (22 ± 2 °C). After removing the solids by centrifugation in ABENCOR equipment (Abengoa, Madrid, Spain), the liquid phase was allowed to decant and the oil was filtered and subjected to analysis. The process is soft [18], minimizes changes in the oily phase during extraction and is well implemented to table olives [6,7,11,19].

For the extraction by a solvent, three weighted aliquots (25 g, 0.1 mg accuracy) of the homogenized paste from the fermented and packaged olives were freeze-dried (Laboratory Freeze-Dryer, Telstar Cryodos) until constant weight. The fat from the solid residue was then separated by extraction with hexane in Soxhlet for 6 h. After removing the solvent in a rotary evaporator at 40 °C, the remaining oil was dried under vacuum in an oven at 60 °C until constant weight [20].

### 2.4. Separation of Polar and Nonpolar Compounds

The oils were fractioned using silica gel columns, according to the procedure developed by Dobarganes, Velasco and Dieffenbacher [21].

### 2.5. Determination of Polar Compounds

The total polar compounds (PC) and their components (polymerized triacylglycerols (PTG), oxidized triacylglycerols (Ox-TG), diacylglycerols (DG), and free fatty acids plus traces of unsaponifiable matter (FFA)) were analysed according to the method developed by Dobarganes et al. [21]. The conditions applied for HPSEC (High-Performance Size Exclusion Chromatography) analysis was as follows: The sample solutions consisted of 10–15 mg of polar compounds/mL in tetrahydrofuran. For separation, an HP1050 system with a 10-µL sample loop and three 50, 100 and 500 Å Ultrastyragel columns (Waters Associates, Milford, MA, USA), 25 cm × 0.77 cm ID, packed with a porous, highly cross-linked styrenedivinylbencene copolymer (<10 µm) connected in series and a refractive index detector (Hewlett-Packard, CA, USA) were used.

### 2.6. Determination of the Unsaponifiable Fraction

The unsaponifiable matter was determined according to the UNE 55004 standard method [22] by saponification of the oil with potassium hydroxide in an ethanolic solution, followed by extraction with diethyl ether.

### 2.7. Determination of Sterols and Triterpenic Dialcohols

These analyses were performed according to the method described in EU No 1348/2013 [9]. The lipid, with added α-cholestanol and betulin as internal standards, was saponified, and the unsaponifiable matter was extracted as mentioned above. The bands corresponding to the sterol and triterpene alcohol fractions were separated from the extract by thin layer chromatography (TLC) on a basic silica gel plate. The sterols, erythrodiol and uvaol recovered from the plate were transformed into trimethylsilyl ethers, and the mixture was analysed by GC using an HP 5890 Series II gas chromatograph equipped with a flame ionization detector and a 30 m × 0.32 mm i.d. Tracsil TRB-5 (95% dimethylpolysiloxane-5% diphenyl, film thickness 0.25 µm) capillary column (Teknokroma, Barcelona, Spain). The chromatographic conditions were injector at 300 °C, isothermal column at 275 °C and detector at 300 °C. The split ratio was 1:50. Hydrogen carrier gas was used at 1.0 mL/min.

### 2.8. Determination of Fatty Alcohols

This analysis was carried out according to the method established in EU No 2015/1833 [23]. The fatty substance, with 1-eicosanol added as an internal standard, was treated as mentioned in the determination of the lipid and unsaponifiable fraction sections. The alcohol fraction was separated from the unsaponifiable matter by chromatography on a basic silica gel plate. The alcohols recovered from the silica gel were transformed into trimethylsilyl ethers and analysed using capillary gas chromatography. The chromatographic conditions were the same as those mentioned above for sterols and triterpenic dialcohols, except that oven temperatures were 215 °C (5 min), 3 °C/min ramp to 290 °C and 2 min. hold. All analyses were performed in duplicate.

### 2.9. Determination of Waxes and Alkyl Esters

The analyses were performed according to the method described in EU No 1348/2013 [9]. In short, they consisted of the addition of two suitably internal standards (lauryl arachidate and methyl heptadecanoate) to the fat and then fractionation by chromatography on a hydrated silica gel column. Recovery under the test conditions of the fraction was eluted first (the polarity of which is less than that of the triacylglycerols); then, direct analysis by capillary column gas chromatography and flame ionization detector followed. Wax esters were quantified using the internal standard lauryl arachidate. Peaks corresponding to esters C36 and C38 were discarded while esters C40–C46 were quantified. Their sum was considered as the wax content. Alkyl esters (methyl and ethyl esters of the C16 and C18 fatty acids) were quantified using methyl heptadecanoate as the internal standard.

### 2.10. Apparatus and Reagents

The determinations were carried out using an HP1050 system (HPLC) equipped with a refractive index detector (Hewlett-Packard, CA, USA) and an HP 5890 Series II gas chromatograph (Hewlett-Packard, MN, USA) fitted with a flame ionization detector.

All reagents were of analytical grade and chromatographic grade.

### 2.11. Statistical Analysis

Data were structured in a matrix array where the rows were samples and the columns were the minor components. Then, the database was checked for normality and outliers.

For conventional analysis, the original data were studied by GLM and the standard multivariate techniques, developed for data in the Euclidean space.

However, according to CoDa, standard multivariate methods were developed for the Euclidean space and are incorrectly applied (at least formally) to the original data in the simplex. Instead, they should be analysed by the methodology specifically developed for its specific sample space, as proposed in this work. Examples of applications in different fields are found elsewhere [6,7,24].

Consequently, when the values were considered as CoDa, which focuses mainly on the relationship among variables, specific exploratory tools like hexatetrahedral plot in the simplex; the bi-plot adapted to CoDa by Aitchison and Greenacre [25]; and the balance dendrogram developed by Pawlowsky-Glahn, Egozcue and Tolosana-Delgado [26] were applied.

The most common transformation into the Euclidean space is the isometric logratio (*ilr*) that moves the original data, row-wise, into the so-called *ilr coordinates*, real vectors in the Euclidean space [27], using the formula:

(1) ilr(J1,J2)=J1·J2J1+J2log((∏j∈J1xj)1J1(∏j∈J2xj)1J2)

where *J*_1_ and *J*_2_ are the numbers of variables in numerator and denominator, respectively.

The standard multivariate methods employed in this work included cluster analysis, based on the Ward method [28] and Principal Component Analysis (PCA), widely used in chemometrics. To obtain the discriminant functions, the backwards stepwise procedure for variable selection was applied (using *p* ≤ 0.05 and *p* ≥ 0.10 for entering and removing, respectively). The minimum tolerance was fixed at 0.00001, and the number of steps was fixed at 100. A leave-one-out cross-validation procedure was adopted to evaluate the performance of the classification rule [16].

MFA deals with data in which a set of individuals is described by several sets of variables [17]. Within each set, variables must belong to the same type, but groups of variables can belong to different categories. As the minor components in the table olive fats are composed of several groups of compounds (fatty alcohols, triterpene dialcohols, sterols, wax, alkyl esters and polar compounds) with several components each, MFA is an appropriate tool for their analysis. MFA performs the first phase of successive PCA by group, followed by a weighted (across column) PCA, and may represent a novel approach to contrast standard multivariate study. Among the advantages of this method are the possibility of studying the components by chemical groups, frequently found in Food Science, and the diversity of statistical options available in the R packages.

Statistica software version 8.0 (StatSoft Inc., Tulsa, OK, USA) was used for GLM, CoDaPack v. 2.01.14 [29] was used for data analysis according to CoDA, XLSTAT version 2017 (Addinsoft, Paris, France) was used for standard multivariate analysis and FactoMinR [30] was used for MFA.

## 3. Results and Discussion

### 3.1. Processing

At the end of fermentation, the brines had the following average values: pH 4.0, titratable acidity 10 g/L, combined acidity 100 mEq/L and NaCl 55 g/L. In the packaged olives, the concentrations of lactic acid and NaCl were 5.5 g /L and 50 g /L, respectively. As the olives followed the usual Spanish-style treatment and had typical levels of the product after processing [2], the changes in the minor components of the Gordal fat (fresh, fermented and packaged fruits) mimicked the transformations that occurred at the industrial scale.

### 3.2. Changes in Total Polar Compounds (PC)

PC, which comprised several degradation products, namely oxidised-triacylglycerols (Ox-TG), diacylglycerols, (DG), and free fatty acids plus a residue of unsaponifiable matter (FFA), constituted only a reduced-fat proportion (1.40–2.07%) (Table 1). The absence of polymerized TG, the strongest oxidation stage [31], means that the Gordal olive fat did not suffer secondary oxidation. Besides, monoacylglycerols (MG) were always below the detection limit. Ox-TG was the most abundant, followed by DG and FFA (Table 1). On the contrary, PC and DG had an overall tendency to increase during processing (fresh fruits vs. fermented or packaged olives) (Table 1). The trend resembled that followed when processing Manzanilla and Hojiblanca cultivars [32]; therefore, it may be characteristic of the green Spanish-style process.

Total PCs in Gordal (Table 1) were of the same order as that in green Spanish-style Manzanilla and Hojiblanca [32] and were within the levels in packaged ripe olives (25–45 mg/g) [11]. PC in the raw material in this work was like that in several Italian cultivars but lower than after their processing as natural green olives [31]. The contents of PC in Gordal also were lower than the concentrations found in extra virgin olive oil (EVOO) with diverse storage periods (approximately 25–33 mg/g) [33]. Hence, the fat from processed Gordal had total PC compatible with EVOO.

Ox-TG, which constitutes an appropriate index for a secondary oxidation degree [31], was the main fraction of the Gordal PC (Table 1). However, highly oxidized products like polymerized Ox-TG, were absent in green Gordal, Manzanilla and Hojiblanca olives [32]. Pasqualone et al. [31] linked the low contents in Ox-TG to their degradation into polymers. In Gordal, the packaging did not affect Ox-TG as with sterilization in ripe olives, although this product reached lower ultimate levels (5.8–7.3 mg/g) [11]. However, other operations like pitting may produce fat residues with high Ox-TG concentrations [34].

The hydrolysis of triacylglycerols leads to DGs [35] strongly related to quality [36]. In Gordal processing, DG only increased after fermentation (Table 1) but led to final contents higher than in other cultivars (3.75–4.15 mg/g oil in Manzanilla and Hojiblanca, respectively) [32]. An enhanced lipolytic activity might have caused the increase of DG in Gordal fermentation because of the favourable predisposition of this cultivar to microbial growth as also observed in ripe olive storage/fermentation (12–30 mg/g oil) where yeasts are usually abundant [11] or in natural green processing [31]. Potential lipolytic microorganisms in emulsified water drops may also be responsible for the high levels of DG in oils from table olive conditioning operations [34].

The concentrations of FFA in the Gordal olives, formed by the hydrolysis of TG or DG [31,35], were low and did not show a systematic trend (Table 1). Their levels in Gordal were unusually low when compared to those found in other Italians natural processes or oils from pitting and stuffing green olives with pepper [31,34].

### 3.3. Effect of Processing on the Unsaponifiable Matter

#### 3.3.1. Sterols

The phytosterols are relevant substances due to their cholesterol-lowering activity [37]. They are usually free (85–90%) [38] and circumstance favourable for analysis. Total sterol in green Spanish-style Gordal was more abundant (2052–3384 mg/kg fat) (Table 1) than in similarly prepared Manzanilla and Hojiblanca (998–1996 mg/kg fat) [32]. The most dominant component was ever β-sitosterol (1867–3085 mg/kg), but the variations in most sterols along Gordal processing were chaotic and only Δ^5^-avenasterol remained stable (Table 1), as also observed in Manzanilla and Hojiblanca cultivars [32] or in a survey on commercial table olives where the differences between cultivars were more important than the changes among presentations [39]. This is a general trend not only in olive elaboration, except for ripe olives [12], but also, likewise, in olive oil extraction.

On the contrary, differences in sterol contents in oils due to cultivars or geographical areas are common [40,41,42]. Sterols played an important role in the establishment of olive oil authenticity because of their specific limits (Table 1, percentage values in parentheses) [9,10]. In this case, cholesterol (just in the limit after some processing phases) and β-sitosterol (always slightly below its threshold) did not fulfil the EVOO requirements.

To notice the many positive significant correlations (not shown) between campesterol, stigmasterol, clerosterol and β-sitosterol with several other compounds, possibly for their parallel formation during maturation but without discounting probable spurious cases due to the quantification method, people use the standard multivariate analysis of sterols for characterization of oils from different olive cultivars or origins [15,41]. Here, standard cluster analysis did not cause efficient segregation since confused GT0 and GT3 as well as GT2 and GT3S and the results using *ilr coordinates* were even worse.

#### 3.3.2. Fatty Alcohols and Triterpene Dialcohols

Olive oil is rich in fatty alcohols with an even number of carbon atoms [8]. The highest overall contents in this work (Table 1) were in Gordal fat extracted by Soxhlet (331–422 mg/kg fat) and the lowest was in the fermented/packaged olives obtained by Abencor (106–157 mg/kg). Their components also had the highest values in samples extracted by Soxhlet, except for docosanol (C_22_). A marked decrease in hexacosanol (C_26_), tetracosanol (C_24_) (except in Hojiblanca) and octacosanol (C_28_) after lye treatment was also observed in Manzanilla and Hojiblanca while docosanol (C_22_) was scarcely affected [32]. Their levels in olive oils were influenced by harvesting date and cultivar [43]. Besides, pomace extraction led to oils with high fatty alcohol contents [44].

The presence of triterpenic pentacyclic dialcohols in olive oils was first reported by Fedeli [45]. In Gordal, the content of erythrodiol was higher than uvaol (Table 1) while uvaol was absent from fermented Gordal fat obtained by Abencor. López-López et al. [12] and Cortés-Delgado et al. [34] also observed the absence of uvaol in oils released from table olive conditioning operations and several phases of ripe olive darkening. The increase in erythrodiol and uvaol, when extracted by Soxhlet, is used to detect pomace oil fraud in olive oil using a specific erythrodiol + uvaol index. It should be ≤4.5% (9, 10), which was overcome in packaged olives regardless of the extraction system (Table 1).

### 3.4. Effect of Processing on the Waxes and Alkyl Esters

The waxes are esters of fatty alcohols and fatty acids in which formation is favoured by triacylglycerol hydrolysis [46]. Most waxes in olive oils have an even number of carbons (C_36_–C_46_). Their contents are higher in pomace oil than in EVOO, a fact used to detect their mixtures [47]. In Gordal, total waxes and their components were low; some of them increased when extracted by Soxhlet but always remained below (Table 1) the thresholds established in the EU regulation for EVOO (≤150 mg/kg) [47]. Wax content was higher in Manzanilla than in Hojiblanca, but processing caused a limited effect (only C_46_ decreased) [32]. However, careless extraction or temperature abuse during olive oil storage increased its level [46].

The reaction of fatty acids with ethanol or methanol produces alkyl esters, which in olive oil, are associated with the bulk storage of olives before extraction [48]. In Spanish-style green olives, the lye treatment produces methanol [49] while fermentation/storage regularly includes ethanol and methanol among their metabolites [2,50]. Lye treatment of Manzanilla and Hojiblanca always raised the levels of methyl esters, but ethyl esters are formed with preference during fermentation [32]. In Gordal, these compounds follow a similar trend (Table 1).

The limit for ethyl esters in EVOO is low (≤35 mg/kg) [47] because high contents contribute consistently to defective notes like fusty, winery, vinegary, muddy sediment, musty [51] or “atrojada” [48]. Conversely, adoption of these compounds to assess the table olive fat quality is not pertinent since their production is a characteristic of fermentation [50]. Alkyl esters (and waxes) were the minor components that exhibited the most systematic increasing trends not only in Gordal (Table 1) but also in Manzanilla and Hojiblanca [32]. Their formation thus calls for particular concern, comprising in-depth fermentation control.

### 3.5. Simultaneous (Multivariate) Study of the Overall Minor Component Changes

**Figure 1:**
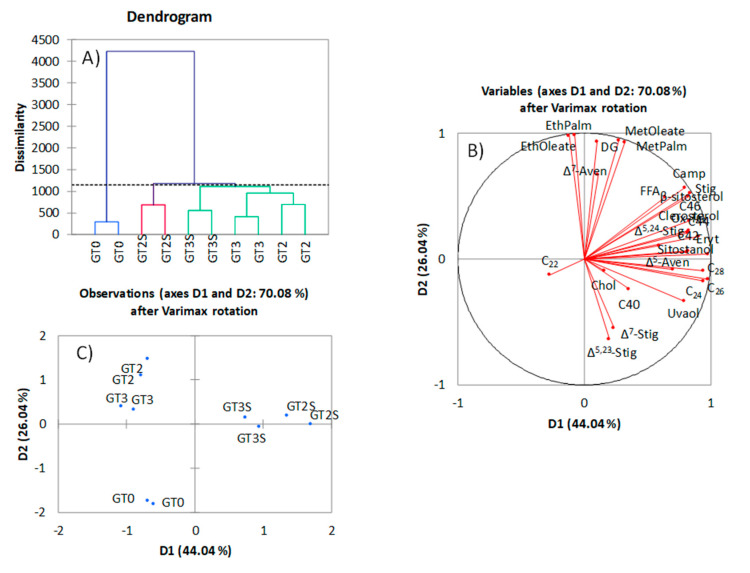
Cluster (**A**) and Principal Component Analysis (PCA) (**B**,**C**), based on the original dataset of the overall minor components in green Spanish-style Gordal table olive fat: GT0, GT2 and GT3 are oils extracted from the fresh, fermented and packaged olives, respectively. Samples coded S were extracted by Soxhlet; otherwise, they were extracted by Abencor. See Table 1 for other abbreviations.

The application of chemometrics to olive oil minor components, expressed in their customary units, is common [11,12,14,15]. Cluster analysis based on the whole matrix of minor components (Figure 1A) led to the formation of three groups (GT0, GT2S and GT3S-GT2-GT3), indicating no distinction in the third group between the fat from the packaged samples extracted by Soxhlet (GT3S) and those form fermented or packaged olives obtained by Abencor (GT2 and GT3). There was a high correlation between methyl and ethyl oleate and palmitate with DG (Figure 1B); that is, formation of the first compounds involves previous hydrolysis of triacylglycerols. They were the sole components strongly linked to D2. ∆^7^-avenasterol was also among them, but as the other components have short rays, it is probable that they were poorly represented on the D1·D2 plane. The remaining parts showed a strong relationship between them and with D1. PCA was efficient for segregation of samples, with D1 (and its associated components) being responsible for the differentiation of samples between extraction systems and D2 (linked to alkyl esters and DG) being responsible for the separation between fresh fruits (GT0), and fermented and packaged products (GT2 and GT3) (Figure 1C). The application of chemometrics based on minor components was also satisfactorily applied for the classification of fat residues from the conditioning process of table olives [16]. Discriminant analysis assigned all samples correctly both in training and cross-validation (data not shown). Then, standard multivariate analysis, using the full dataset of the minor components in usual units, was reasonably efficient in segregating fats from the various processing steps, although it led to a relatively close position between fermented (GT2) and packaged (GT3) olives. However, the EVOO legislation completely obviates consideration of overall minor components for classification [47]. 

**Figure 2:**
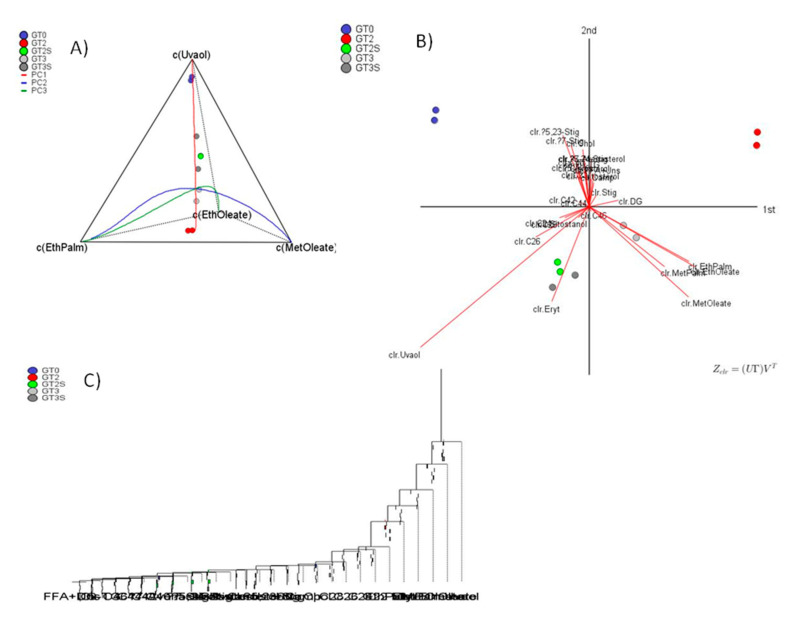
Changes in the minor components of the green Spanish-style Gordal table olive fat, according to processing phases and extraction methods, using specific exploratory tools for compositional data analysis: (**A**) tetrahedral plot in the simplex as a function of compounds with the highest logratio variability, (**B**) form biplot and (**C**) balance dendrogram. GT0, GT2 and GT3 are oils extracted from the fresh, fermented and packaged olives, respectively. Samples coded S were extracted by Soxhlet; otherwise, they were extracted by Abencor. See Table 1 for other abbreviations.

**Figure 3:**
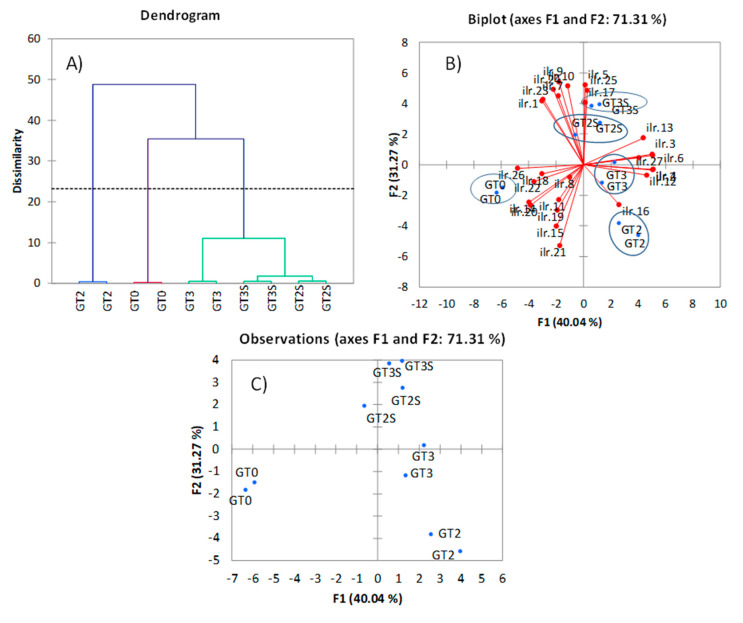
Cluster (**A**) and PCA (**B**,**C**) based on *ilr coordinates* of the overall original minor components’ data in green Spanish-style Gordal table olive fat: GT0, GT2 and GT3 are oils extracted from the fresh, fermented and packaged olives, respectively. Samples coded S were extracted by Soxhlet; otherwise, they were extracted by Abencor. *ilr* 1–25 stand for the successive *coordinates* obtained by isometric logratio transformation.

CoDa analysis of all minor components showed that uvaol, methyl oleate, ethyl oleate and ethyl palmitate were the components with the highest values in the variance array. The tetrahedral plot (Figure 2A) displayed a marked difference between GT0 and processed fruits, with the fat from fermented olives (GT2) being the most different. Interestingly, the changes followed the direction of PC1, showing a linear trend. The form biplot (Figure 2B) was segregated between GT0, GT2, and GT3 (all of them obtained by Abencor) but not between GT2S and GT3S (extracted by Soxhlet). In the dendrogram (Figure 2C), the balances (always the logratio of the component on the right of the horizontal axis over the geometric mean of those remaining on its left) progressively accounted for lower variances, which were 35.718 (uvaol), 11.07 (ethyl oleate), 12.603 (methyl oleate) and 12.276 (methyl palmitate). The remaining balances had variances <1, indicating that, for CoDa, they were scarcely affected by processing. These balances formed (row-wise) the matrix of the *ilr coordinates.* Application of the multivariate tools to original data transformed into *ilr coordinates* segregated three clusters, GT0, GT2 and GT3-GT2S-GT3S, as in the analysis with the original data set. However, the greatest dissimilarity in this case was observed for the fat from fermented olives (GT2) extracted by Abencor. The fresh olives (GT0) also retained their particular characteristics, and within the third group, those extracted by Soxhlet (GT2S and GT3S) were considered different from packaged olives, although at a nonsignificant level (Figure 3A).

In comparison to the clustering distribution obtained with the original units, the groups based on *ilr coordinates* were more reasonable since associated samples were extracted by Soxhlet and identified fermented olives as a highly different group. This distinction could agree to the sensible transformation that olives suffer during fermentation [2]. The standard biplot based on *ilr coordinates* (Figure 3B) and, mainly, PCA (Figure 3C) differentiated samples according to processing phases and extraction system. In both cases, D1 separated fresh from processed fruits. At the same time, D2 was segregated, extracted by Abencor (on its negative side), from those by Soxhlet (on the positive), with a further separation between fermented (mainly) and packaged olives. Identification of the compounds responsible for discrimination in CoDa PCA was problematic owing to the complexity of their definitions. DA based on *ilr coordinates* only assigned the samples correctly in training processes but failed in cross-validation. The sound grouping observed in clustering and PCA by using CoDa analysis is also an interesting favourable effect of CoDa analysis, although their causes are still unknown since *ilr* coordinates should reproduce the original data structure. Then, this work demonstrates that CoDa analysis, which was already used satisfactorily for the study of fatty acids [6], can be extended similarly to the minor components not only of table olives but also of olive oil.

**Figure 4:**
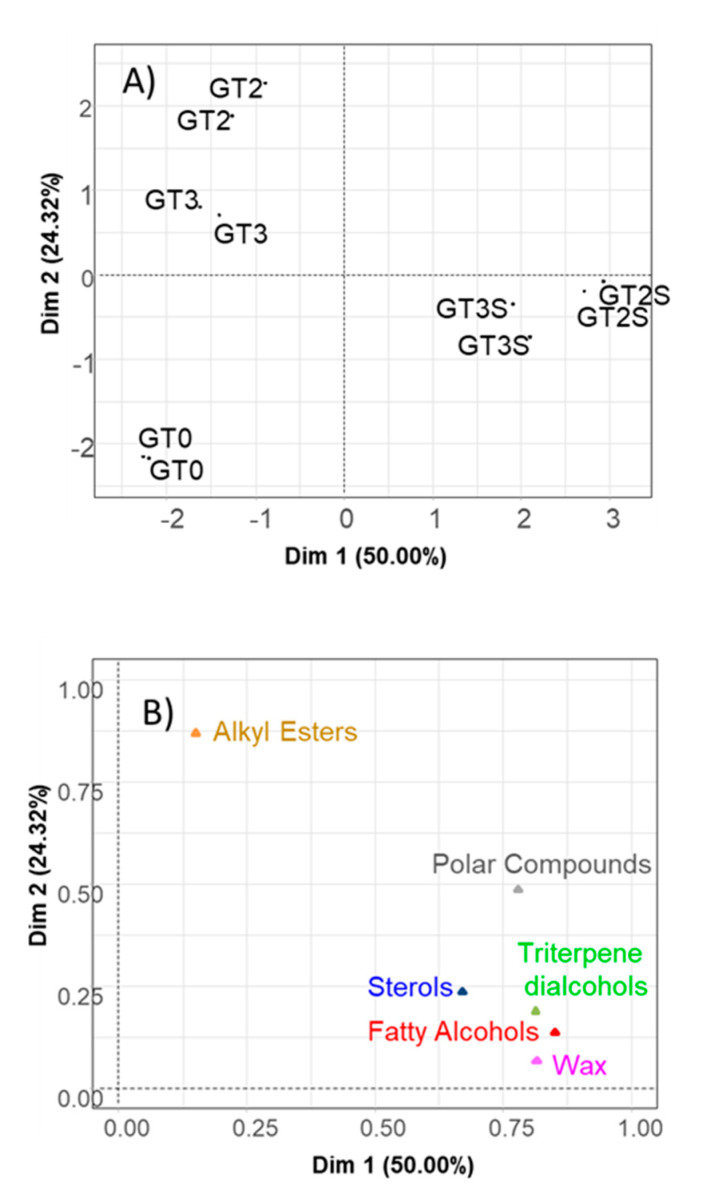
A study, based on the original data, of the minor components’ changes in green Spanish-style Gordal fat according to processing phases and extraction method using Multiple Factor Analysis (MFA): position of the samples (**A**) and groups (**B**) of compounds in the factor map. GT0, GT2 and GT3 are oils extracted from the fresh, fermented and packaged olives, respectively. Samples coded S were extracted by Soxhlet; otherwise, they were extracted by Abencor.

For MFA, the groups considered were fatty alcohols, triterpene dialcohols, sterols, wax, alkyl esters and polar compounds. The components included in each of them were those already mentioned in previous sections. The first two dimensions of MFA explained 50.00 and 24.32% (jointly, 74.32%) of the variance and, then, described most of the data structure. D1 segregated samples according to the extraction system, while D2 separated fresh fruits from fermented and packaged when extracted by Abencor (Figure 4A). Then, MFA was somewhat similar to standard PCA, although with a slight improvement in segregation and closeness of replicates even compared to the PCA on *ilr coordinates* (CoDa) and with a better separation between fat samples from fermented and packaged olives extracted by Abencor. Most of the groups of components (Figure 4B) were strongly associated to D1, showing their predominant link to samples obtained by Soxhlet (Figure 4A), while polar and alkyl esters (mainly) were more associated with D2 (Figure 4B), and fermented and packaged samples extracted by Abencor (Figure 4A). The specific position of alkyl esters is in accordance with its production during processing. Then, this analysis allows not only the application of standard multivariate techniques [12,14,39] (among others) but also more detailed information on the compounds of similar characteristics.

**Figure 5:**
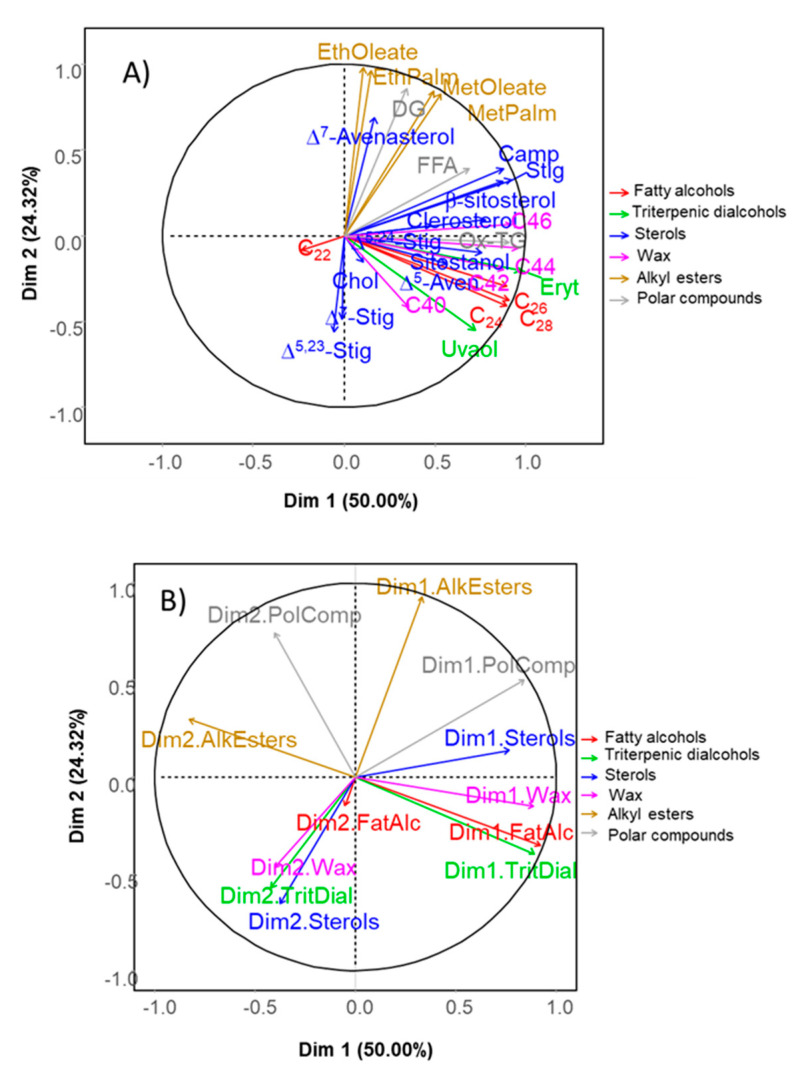
A study, based on the original data of the changes, according to processing phases and extraction method in the minor components in green Spanish-style fat using Multiple Factor Analysis (MFA): a correlation circle for groups and individual components (**A**) and between the first two dimensions of MFA and PCA of the groups’ components (**B**). See Table 1 for abbreviations.

Although a first approach to the relationship between groups was previously noticed (Figure 4B), the RV coefficient, which also ranges from 0 to 1, allows for quantification of their correlations (Table 2). The proximity between sterols, fatty alcohols, triterpene dialcohols and waxes suggests close similar metabolism pathways and a great dependency of their levels in fat on the extraction system. Conversely, polar compounds and alkyl esters are more related to processing. The links between groups and compounds within them are envisioned in the circle of correlation (Figure 5A), which includes the individual compounds and groups. As in the standard PCA, cholesterol, Δ^7^-stigmasterol and Δ^5,23^-stigmastadienol might again not be fully described in this case (short arrows), indicating a remarkable similarity between statistical procedures. The strong correlation between most groups, on the one side, and alkyl esters and DG, on the other, agrees to Figure 5B.

As mentioned, MFA starts with a PCA by groups. The agreement between these PCA dimensions and those of MFA may be an indicator of the representativeness of the second regarding the overall groups. Most of the individual PCA first (second) dimensions are related to the MFA first (second) dimensions, with only a slight shift to the left (Figure 5B). However, in the case of polar compounds and alkyl esters (mainly), the turn should be in the opposite direction, revealing, again, their peculiarities. Therefore, MFA can represent the data structure of the minor components well, even preserving the alkyl esters and DG peculiarities. However, the application of just PCA, although it was able to separate olive samples according to their cultivar and growing area [15] based on fatty acids, and sterols did not study the influence of groups of compounds.

**Figure 6:**
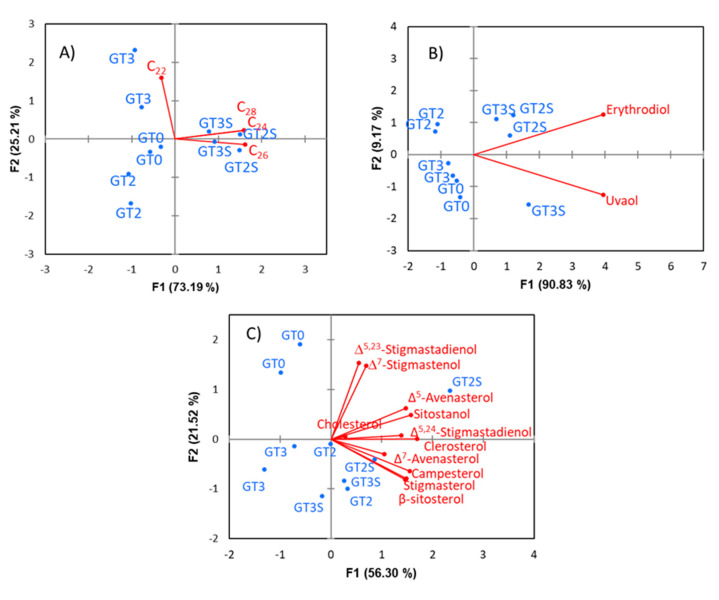
A study, based on the original data, of the changes according to processing phases and extraction method in the minor components in green Spanish-style fat using Multiple Factor Analysis (MFA): PCA was done according to minor component groups. Fatty alcohols (**A**), triterpene dialcohols (**B**) and sterols (**C**). GT0, GT2 and GT3 are oils extracted from the fresh, fermented and packaged olives, respectively. Samples coded S were extracted by Soxhlet; otherwise, they were extracted by Abencor.

**Figure 7:**
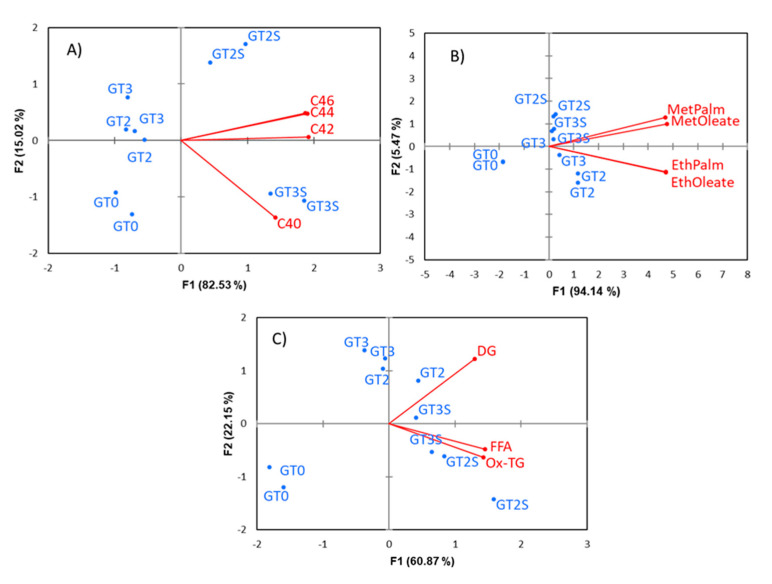
A study, based on the original data of the changes, according to processing phases and extraction method in the minor components in green Spanish-style fat using Multiple Factor Analysis (MFA): PCA was done according to minor component groups. Waxes (**A**), alkyl esters (**B**) and polar compounds (**C**). GT0, GT2 and GT3 are oils extracted from the fresh, fermented and packaged olives, respectively. Samples coded S were extracted by Soxhlet; otherwise, they were extracted by Abencor. See Table 1 for abbreviations.

However, the relationship between the individual components of each olive group with processing and extraction methods was explained by their particular PCA, performed previously for MFA. There was a clear trend in separation alongside D1 according to the extraction method due, mainly, to fatty alcohol (Figure 6A), triterpene dialcohols (Figure 6B), sterols (Figure 6C) and waxes (Figure 7A). Nevertheless, alkyl esters (Figure 7B) were segregated between fresh and processed fruits without the influence of the extraction method. Possibly other components like C22 (Figure 6A) and diglycerols (Figure 7C), together with slight trends in some compounds from other groups, also contributed to further separation between fermented (slightly more abundant in alkyl esters) and packaged olives.

**Figure 8:**
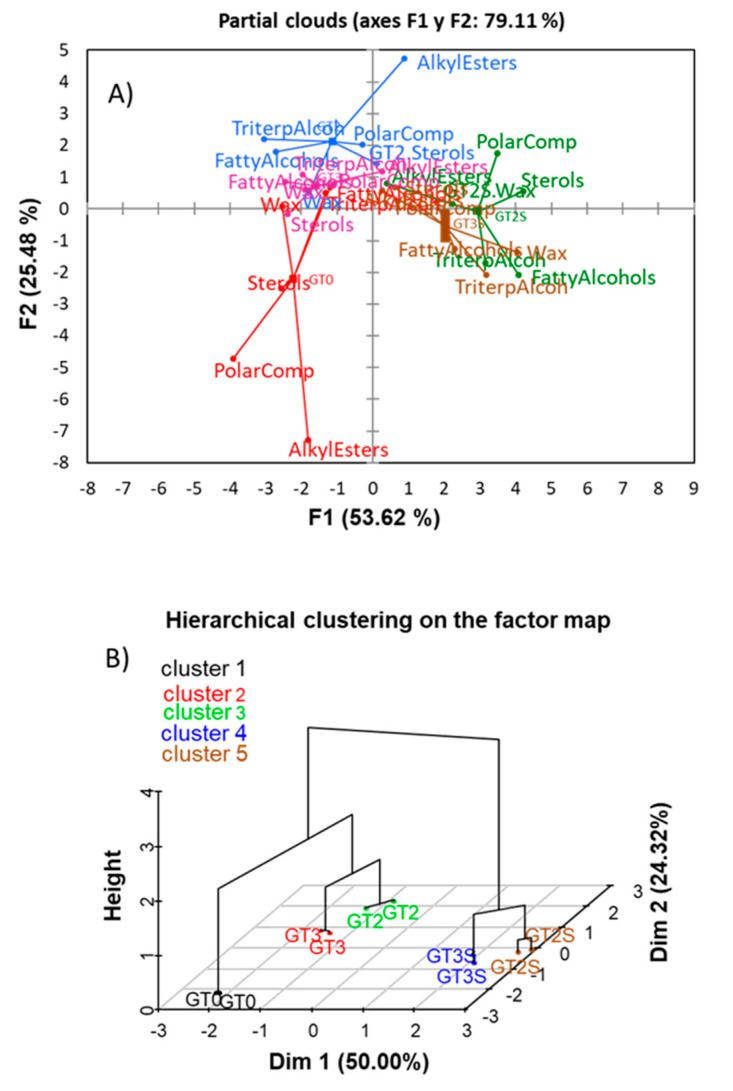
A study, based on the original data, of the changes according to processing phases and extraction method in the minor components in green Spanish-style fat using Multiple Factor Analysis (MFA): PCA was done according to minor component groups. Partial clouds were according to groups of compounds (**A**), and hierarchical clustering was done on a factor map (**B**). GT0, GT2 and GT3 are oils extracted from the fresh, fermented and packaged olives, respectively. Samples coded S were extracted by Soxhlet; otherwise, they were extracted by Abencor. See Table 1 for other abbreviations.

The effect of different groups of compounds on the position of the centre of the samples (to facilitate visualization) was summarized in Figure 8A. One can see that decreasing the content in alkyl esters (mainly) and polar compounds leads to further separation of fresh from processed fruits. Besides, the increment on polar compounds, sterols and fatty alcohols could separate the extracted-by-Soxhlet samples (particularly GT2S) from the fermented and packaged samples.

Similarly, alkyl ester increase improves separation of fermented fruits from fresh olives. Finally, the correct grouping of samples, according to processing phases and the proximity of the replicates, after hierarchical clustering on the plane of the samples (Figure 8B) should be emphasized. MFA was not applied to *ilr coordinates* because of the problematic interpretation of the results.

Finally, a concise comparison among some characteristics of the multivariate methods used in the work is shown in Table 3. From it, one can deduce that a combination of CoDa analysis (for working in the simplex) and MFA (to take advantages of their numerous options) could be an exciting challenge.

## 4. Conclusions

This work presents a detailed classical study of each minor component change during processing and extraction. Still, the multivariate analysis provided a straightforward interpretation and showed that processing, particularly the fermentation phase, affected mainly alkyl esters and diacylglycerols. At the same time, extraction by Soxhlet affected the fatty alcohols, triterpene dialcohols, waxes and polar compounds’ concentrations.

Besides, it has demonstrated that, apart from the conventional and traditional multivariate statistical study of minor components in olive fat, other alternatives are viable, like CoDa and MFA. The results obtained by standard multivariate and applying CoDa were relatively similar, indicating that CoDa concepts can be satisfactorily useful for studying olive oil minor components, with the advantage of working in an appropriate sampling space: the simplex. MFA led to a highly informative multivariate study and particularly accurate hierarchical clustering; however, its use with *ilr coordinates* resulted in difficult interpretation. Possibly, basing its implementation on other logratio transformations could be an exciting challenge.

## Figures and Tables

**Table 1:** Distribution of the green Spanish-style Gordal table olive fat between polar and nonpolar compounds as well as its concentration in minor components, according to processing phases and extraction method.

Compound	Abencor Extraction			Soxhlet Extraction			
	Fresh	Fermented	Packaged	Fermented	Packaged	SE *	Limits
	Polar and Non-Polar Compounds in Table Olive Fat (%)						
Polar	1.40 ^b^	2.07 ^a^	2.02 ^a^	1.98 ^a^	1.96 ^a^	0.03	
Non-polar	95.16 ^a^	94.63 ^b^	93.95 ^c^	95.52 ^a^	94.06 ^c^	0.09	
Total	96.6 ^b^	96.7 ^b^	96.0 ^c^	97.5 ^a^	96.0 ^c^	0.1	
	Polar compounds (mg/g oil)						
Ox-TG	9.1 ^b^	10.0 ^a^	9.2 ^b^	10.1 ^a^	9.4 ^b^	0.2	
DG	2.7 ^c^	6.7 ^b^	7.7 ^a^	7.0 ^b^	7.8 ^a^	0.2	
MG	Nd	Nd	Nd	Nd	Nd	N/A	
FFA	2.2 ^c^	4.0 ^a^	3.3 ^b^	2.7 ^c^	2.4 ^c^	0.1	
Total PC	14.0 ^c^	20.7 ^a^	20.2 ^a^	19.8 ^b^	19.6 ^b^	0.2	
	Sterols (mg/kg)						
Cholesterol	10.3 ^a^(0.5%)	11.1 ^a^(0.4%)	5.8 ^b^(0.3%)	8.4 ^a b^(0.3%)	11.1 ^a^(0.5%)	0.8	≤0.5%
Campesterol	46 ^d^(2.2%)	68 ^b^(2.4%)	54 ^c^(2.5%)	82 ^a^(2.4%)	70 ^b^(3.9%)	2	≤4.0%
Stigmasterol	21 ^d^	37 ^b^	28 ^c^	49 ^a^	41 ^b^	1	<camp
Δ^5,23^-Stigmastadienol	10.5 ^a^	5.8 ^b^	4.9 ^b^	9.6 ^a^	4.3 ^b^	0.9	
Clerosterol	29 ^b c^	33 ^b^	26 ^c^	42 ^a^	33 ^b c^	2	
β-sitosterol	1867 ^c^(91.0%)	2564 ^b^(92.1%)	2009 ^c^(91.8%)	3085 ^a^(91.2%)	2606 ^b^(91.8%)	82	≥93.0%
Sitostanol	12 ^b^	12 ^b^	17 ^a b^	35 ^a^	24 ^a b^	6	
Δ^5^-Avenasterol	27 ^a^	26 ^a^	23 ^a^	41 ^a^	28 ^a^	5	
Δ^5,24^-Stigmastadienol	8 ^a b^	10 ^a b^	7 ^b^	12 ^a^	8 ^a b^	1	
Δ^7^-Stigmastenol	14 ^a^(0.3)	10 ^a^(0.3)	8 ^b^(0.3)	14 ^a b^(0.2)	8 ^b^(0.2)	2	≤0.5%
Δ^7^-Avenasterol	6.1 ^b^	7.5 ^a^	6.3 ^b^	6.9 ^a b^	6.4 ^b^	0.3	
Total	2052 ^c^	2784 ^b^	2189 ^c^	3384 ^a^	2840 ^b^	90	≥1000
	Fatty alcohols and triterpenic dialcohols (mg/kg)						
Docosanol (C_22_)	35 ^b^	25 ^b^	54 ^a^	32 ^b^	35 ^b^	4	
Tetracosanol (C_24_)	11.2 ^b^	6.7 ^c^	10.2 ^b^	21.3 ^a^	20.4 ^a^	0.5	
Hexacosanol (C_26_)	81 ^c^	33 ^d^	29 ^d^	227 ^a^	172 ^b^	5	
Octacosanol (C_28_)	62 ^c^	42 ^d^	63 ^c^	141 ^a^	104 ^b^	5	
Total fatty alcohols	189 ^c^	106 ^d^	157 ^d^	422 ^a^	331 ^b^	5	
Erythrodiol	23 ^c^	18 ^c^	189 ^a^	20 ^c^	164 ^b^	6	
Uvaol	6 ^a b c^	0 ^c^	11 ^a b^	4 ^a c^	13 ^a^	2	
Erythrodiol +Uvaol	29 ^b^	18 ^b^	200 ^a^	25 ^b^	177 ^a^	7	
Eryt+Uv (EU) (%)	1.4 ^b^	0.6 ^b^	5.6 ^a^	1.1 ^b^	5.9 ^a^	0.3	
	Waxes (mg/kg)						
C40	16.0 ^b^	13.9 ^b^	12.6 ^c^	13.8 ^b^	21.4 ^a^	0.7	
C42	18.7 ^c^	18.5 ^c^	21.5 ^b^	30.0 ^b^	36.3 ^a^	0.7	
C44	16 ^b^	18 ^b^	18 ^b^	31 ^a^	32 ^a^	1	
C46	1.9 ^b^	2.8 ^b^	2.5 ^b^	4.5 ^a^	4.8 ^a^	0.4	
C42+C44+C46	36.0 ^c^	39.1 ^c^	41.4 ^c^	65.2 ^b^	72.6 ^a^	0.7	
Total	52 ^c^	53 ^c^	54 ^c^	79 ^b^	94 ^a^	3	
	Alkyl esters (mg/kg)						
Methyl palmitate	1.4 ^c^	19.2 ^a^	16.1 ^b^	18.3 ^a^	16.0 ^b^	0.3	
Ethyl palmitate	1.3 ^d^	33.6 ^a^	20.4 ^b^	16.8 ^c^	19.1 ^b c^	0.9	
Methyl oleate	3 ^c^	90 ^a^	71 ^b^	77 ^b^	75 ^b^	1	
Ethyl oleate	4 ^d^	126 ^a^	84 ^b^	66 ^c^	64 ^c^	5	
Total	10 ^c^	268 ^a^	192 ^b^	178 ^b^	174 ^b^	6	

Notes: * SE, pooled standard error according to compounds; Ox-TG, Oxidized triacylglycerols; DG, Diacylglycerols; MG, Monoacylglycerols; FFA, Free fatty acids + unsaponifiable traces; PC, Polar compounds. Data in each cell are the average (unweighted mean) of two samples per replicate. Values within rows followed by different letters are different at *p* ≤ 0.05. Values in parenthesis are the percentages that represent each sterol in fat from the different processing phases and extraction methods. Limits, legal limits established by the IOC for EVOO.

**Table 2:** Coefficient RVs for the correlation between the different groups used for Multiple Factor Analysis (MFA) of minor compounds in the fat from green Spanish-Style Gordal table olives according to processing phases and fat extraction method.

	Fatty Alcohols	Triterpene Dialcohols	Sterols	Waxes	Alkyl Esters	Polar Compounds	Multiple Factor Analysis (MFA)
Fatty alcohols	1.000	0.861	0.538	0.654	0.055	0.496	0.805
Triterpene dialcohols	0.861	1.000	0.401	0.745	0.086	0.462	0.790
Sterols	0.538	0.401	1.000	0.368	0.356	0.642	0.747
Waxes	0.654	0.745	0.368	1.000	0.119	0.586	0.772
Alkyl esters	0.055	0.086	0.356	0.119	1.000	0.641	0.503
Polar compounds	0.496	0.462	0.642	0.586	0.641	1.000	0.858
Multiple Factor Analysis (MFA)	0.805	0.790	0.747	0.772	0.503	0.858	1.000

**Table 3:** Main characteristics of the standard multivariate statistics, compositional data (CoDa) and multifactor analysis.

Standard Multivariate Techniques	CoDa	Multifactor Analysis
General: - Developed for the Euclidean space. - Applicable to interval scale data. - The methods are always applied to data in the original units. - Use with CoDa presents numerous drawbacks (negative correlations, covariance dependence on parts, singular variance matrices, nonnormal distribution, etc.). In this work: - Clustering and PCA analyses led to apparent reasonably results.	General: - For analysis in the simplex. - Techniques specific for CoDa. - CoDa are multivariate by nature and only carry relative information. - CoDa is permutation invariant and subcompositional coherent and shows subcompositional dominance. - CoDA analyses are performed by stay-in-the-simplex methods or by standard multivariate tools after transformation into the Euclidean space. In this work: - Data were analysed by several powerful exploratory tools (biplot, variation array and dendrogram) specific for CoDa. - CoDa transformed into *ilr coordinates* (Euclidean real space) were also subjected to standard multivariate techniques. - Using *ilr coordinates*, standard clustering and PCA led to better results than with standard techniques using the original data.	General: - For analysis in the Euclidean space. - Applicable to interval scale data. - Similar problems than the standard multivariate tools when applied to CoDa. - MFA may apply to data grouped into chemical families. - Packages available in R provide numerous options and outputs. In this work: - MFA allowed data analysis by groups of components, a study of the correlation between them and the evaluation of chemical groups’ effects on samples. - PCA dimensions were defined as a function of the individual parts and based on chemical groups. - Led to more realistic clustering and distribution of samples on PCA than the other standard and CoDa alternatives. - When applied to *ilr coordinates*, the interpretation of results was difficult. The implementation of other logratios could allow new CoDa options.
